# Acute effects of various doses of nitrate-rich beetroot juice on high-intensity interval exercise responses in women: a randomized, double-blinded, placebo-controlled, crossover trial

**DOI:** 10.1080/15502783.2024.2334680

**Published:** 2024-03-27

**Authors:** Zhang Jiaqi, Dai Zihan, Stephen Heung-Sang Wong, Zheng Chen, Eric Tsz-Chun Poon

**Affiliations:** a The Chinese University of Hong Kong, Department of Sports Science and Physical Education, Hong Kong, Shatin, New Territories, China; b The Educational University of Hong Kong, Department of Health and Physical Education, Hong Kong, Tai Po, New Territories, China

**Keywords:** Nitrate, beetroot juice, interval exercise, ergogenic aid

## Abstract

**Background:**

This study investigated the acute effects of various doses of nitrate-rich beetroot juice on the responses to high-intensity interval exercise in women.

**Methods:**

A double-blinded, randomized, placebo-controlled, crossover trial was conducted with 13 recreationally active young women (age = 23 ± 2 years). All participants performed interval exercise (8 × 1-min bouts of cycling at 85% of peak power output [PPO] interspersed with 1-min active recovery at 20% of PPO) 2.5 h after consumption of the randomly assigned beetroot juice containing 0 mmol (placebo), 6.45 mmol (single-dose), or 12.9 mmol (double-dose) ${NO}_{3}^{-}$. The heart rate (HR), blood pressure, blood lactate, blood glucose, oxygen saturation, rating of perceived exertion (RPE), and emotional arousal were assessed.

**Results:**

Nitrate supplementation significantly altered the HR and RPE responses across the three trials. The mean HR was lower in the single- and double-dose groups than in the placebo control group during both work intervals and recovery periods, as well as across the overall protocol (all *p* < .05). The mean RPE was lower in the single- and double-dose groups than in the control group during recovery periods and across the overall protocol (all *p* < .001). However, there was no significant difference in either HR or RPE between the single- and double-dose groups at any time point.

**Conclusions:**

Acute nitrate ingestion led to significant decreases in the mean HR and RPE during high-intensity interval exercise, but no additional benefit was observed with higher nitrate content. These findings may assist practitioners in implementing more effective nitrate supplementation strategies during high-intensity interval exercise.

## 1. Introduction

Nitric oxide (NO) is a crucial signaling and regulatory molecule in the human body that is involved in various physiological processes such as vasodilation [1], mitochondrial respiration [2], skeletal muscle contractility [3], and the development of fatigue [4]. The human body has two complementary pathways to generate NO, the NO synthase (NOS)-dependent pathway (i.e. the biosynthesis of NO from the conversion of L-arginine to L-citrulline in the presence of oxygen) and the nitrate-nitrite-NO pathway, which requires a series of intricate inter-organ reactions [5–7]. Notably, dietary consumption of green leafy or root vegetables such as spinach, kale, carrots, and beetroot, accounts for a significant portion of the nitrate (${NO}_{3}^{-}$) involved in the latter pathway (~80%) [8,9]. Owing to the unique role of NO in improving mitochondrial efficiency [10] and muscle contractile efficiency [11–14] during exercise, over the past decade, dietary nitrate consumption has been extensively studied in nutritional research for its potential benefits on health and exercise performance. In particular, the latest International Olympic Committee consensus statement [15] suggests that nitrate supplementation is associated with improvements of 4–25% in time-to-exhaustion exercise performance and 1–3% in time-trial performance lasting <40 min in duration [16,17].

While numerous studies have investigated the effects of nitrate supplementation on exercise performance, the majority of research has focused on endurance-based exercise protocols, such as continuous running [18–20], cycling [21–27], and rowing [28,29]. However, in recent years, the research focus has been shifted to high-intensity interval exercise [30–35], which is characterized by alternating periods of intense effort and recovery [36]. This exercise regimen has gained widespread popularity in recent years as an effective and time-efficient training modality for improving cardiovascular fitness and performance in various populations [37]. Nonetheless, most studies with interval exercise protocols completed to date [30–33,35] applied chronic nitrate supplementation, with only one study [34] examining the acute ingestion of nitrate. Therefore, the acute effects of dietary nitrate supplementation on physiological and psychological responses to interval exercise should warrant further investigation.

In addition, the optimal dose of nitrate supplementation to maximize performance benefits remains unclear, as previous studies have utilized a wide range of nitrate doses. For example, one study reported that performance measured by distance covered in the Yo–Yo Intermittent Recovery Test Level 1 test was improved by 3.4–14% following nitrate supplementation [38–40], with the greatest improvement observed with an acute dose of 12.9 mmol ${NO}_{3}^{-}$ [38]. In contrast, another study by Bender et al. [34] found no improvement in either peak power output or mean power output after acute ingestion of 12.9 mmol ${NO}_{3}^{-}$ in recreationally active men. Additionally, no differences were observed in heart rate (HR) or blood pressure. These findings were consistent with those of Wylie et al. [30], in which chronic supplementation of the same dosage (i.e. 12.9 mmol ${NO}_{3}^{-}$/d) was applied. However, when participants were given a chronic nitrate supplementation at a smaller dose, total work was improved by 22.3% [33].

Another notable limitation of existing research is the predominant focus on men, which may have overlooked potential sex differences in responses to nitrate supplementation [41]. A recent systematic review [41] revealed that of 123 studies examining the effectiveness of nitrate supplementation, only seven studies specifically recruited women. Furthermore, the effects of acute nitrate consumption on an exclusively recreationally active women population have been insufficiently studied [42]. It is well-known that hormone profiles differ between men and women [43]. In terms of sex hormone composition, men generally have higher testosterone values compared to women, while women have higher estrogen values than men. Consequently, men tend to have greater skeletal muscle mass due to the anabolic effect of testosterone, which directly stimulates muscle protein synthesis [43]. Since skeletal muscle also serves as the storage reservoir for ${NO}_{3}^{-}$and ${NO}_{2}^{-}$ [44], the disparity in muscle mass between men and women may affect impact the storage, utilization, and retention of nitrates following supplementation. Moreover, sex-associated difference in resting blood pressure, as indicated by lower resting blood pressure in young women than in men of similar age, is speculated to be related to estrogen levels [45]. This disparity in baseline values, may result in different lowering effect of nitrate supplementation on blood pressure. Despite these potential sex differences, women remain underrepresented as research participants in this research field. Therefore, it is crucial to include women in investigations on the effects of nitrate-rich beetroot juice during exercise.

Given that the majority of existing literature has primarily focused on endurance-based exercise protocols, applied various nitrate doses, and exhibited a predominant interest in men, the current study aimed to investigate the acute dose–response effects of nitrate supplementation by utilizing a high-intensity interval exercise protocol and recruiting women participants. We hypothesized that a higher dose (12.9 mmol) of acute ${NO}_{3}^{-}$ intake would induce greater physiological responses as indicated by HR, blood pressure, blood glucose, lactate, and oxygen saturation, as well as psychological responses as indicated by RPE and emotional arousal during high-intensity interval cycling than a lower dose (6.45 mmol) and placebo control (0 mmol).

## 2. Materials and methods

### 2.1. Participants

Thirteen healthy, recreationally active women with a regular menstrual cycle (age = 22.9 ± 1.8 years, body mass = 56.4 ± 6.4 kg, BMI = 21.1 ± 1.9 kg/m^2^, and peak power output [PPO] = 123 ± 25 W) enrolled voluntarily in this study. Participants were included if they 1) had a BMI between 18.5 and 24.9 kg/m^2^ [46] and 2) met the requirement of moderate level of physical activity per week (i.e. equivalent to 150 min of moderate intensity physical activity performed per week), as assessed by the International Physical Activity Questionnaire [47]. Participants were excluded if they 1) had blood pressure ≥130/80 mmHg; 2) took medication that affects vasodilation, heart rate, or stomach acid production; 3) took anti-coagulant medications; 4) took oral contraceptive pills; 5) had orthopedic limitations; 6) had a diagnosis of cardiovascular disease or Type I or II diabetes; or 7) had a history of myocardial infarction, uncompensated heart failure, or unstable angina pectoris [48,49].

During the screening process, the participants were informed of the experimental procedures, provided written, informed consent, and completed health history and physical activity questionnaires. The study protocol adhered to and respected the tenets of the Declaration of Helsinki and was approved by the Survey and Behavioral Research Ethics Committee of the Chinese University of Hong Kong (Reference No. SBRE-21-0511. Date of approval: 15 February 2022).

### 2.2. Experimental design

**Figure 1.**
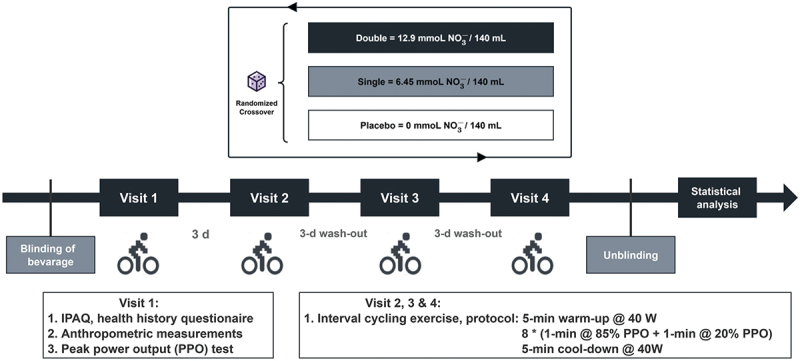
Overview of the experimental protocol and methodological aspects of study timeline.

This was a randomized, double-blind, placebo-controlled, crossover trial. After recruitment, the participants reported to the laboratory on four separate occasions over 11 days. Visit 1 was a preliminary test for anthropometric measurements and the PPO test. During visits 2, 3, and 4, the participants arrived at the laboratory in a rested state (abstinence from strenuous exercise 24 h preceding the trial) in the morning after an 8-h overnight fast and performed interval exercise 2.5 h after the consumption of the randomly assigned dose of beetroot juice with a standard meal. A washout period of 72 h was used to separate each visit to allow for sufficient recovery, in line with previous research investigating the acute effects of beetroot juice supplementation [50,51]. A maximum washout period of up to 6 days was allowed under special circumstances (e.g. illness). All trials were performed at the same time of day (±1 h) and under controlled environmental conditions (20ºC–22ºC and 30–36% humidity). Participants were asked to record their food intake 24 h prior to the first main trial and replicate the same diet in the 24 h preceding all subsequent trials. They were also required to refrain from eating foods or drinks with caffeine or a high nitrate content 24 h prior to each trial, as well as antibacterial mouthwash and chewing gum for the entire study duration, as they would attenuate the reduction of nitrate to nitrite in the oral cavity [52]. Furthermore, the participants were asked to report any gastrointestinal intolerance or side effects from consuming the supplementation at the end of the last experimental trial. The overall study design is illustrated in Figure 1.

Double = double-dose group; Single = single-dose group; Placebo = placebo control group; IPAQ, International Physical Activity Questionnaire.

### 2.3. Supplementation procedures

The nitrate-rich beetroot juice (batch number: 1K30) and placebo beverage (batch number: 2A07) employed in the present study were commercially available and purchased from Beet It Sport, James White Drinks Ltd. (Suffolk, England, United Kingdom). The company consistently provided nitrate content within the manufacturer’s specifications and normally tested random batches to a third-party testing service provider for nitrates levels. Both beetroot juices were made with the same ingredients: 98% concentrated beetroot juice plus 2% concentrated lemon juice. The nutrients in the placebo shots were the same as the standard ones with the only difference in nitrate concentrations. In placebo beverages, nitrates are extracted using an ion-exchange resin while maintaining the same taste, appearance (color), and macronutrients. A previous study examining the nitrate content of beetroot juice and placebo beverages confirmed the product integrity [53]. To ensure blinding, the nitrate-rich beetroot juice and placebo beverage were packaged with number labels by and independent research student.

During visits 2, 3, and 4, the participants arrived at the laboratory under fasting conditions. They randomly consumed either 2 × 70 mL nitrate-rich beetroot juice (double-dose group, ~12.9 mmol ${NO}_{3}^{-}$), 70 mL nitrate-rich beetroot juice +70 mL placebo beverage (single-dose group, ~6.45 mmol ${NO}_{3}^{-}$), or 2 × 70 mL placebo beverage (placebo control group, ~0.0068 mmol ${NO}_{3}^{-}$) along with a standard meal (one portion of scrambled egg, one chicken steak, and one piece of toast). The total caloric intake of the meal (including the beverage) was 771.2 kcal, which consisted of 42.2 g of carbohydrates, 46 g of fat, and 42.2 g of protein. The caloric value is calculated by a registered dietitian using a local government food database (https://www.cfs.gov.hk/english/nutrient/fc-introduction.php). During the postprandial period, the participants rested in the lab for 2.5 h and were allowed to consume only plain water.

### 2.4. PPO test

During visit 1, all participants completed an incremental exercise test on an ergometer bike (LC7, Monark, Sweden) at a constant, self-selected pedal rate (60–80 rpm). The seat height was appropriately adjusted, recorded, and reproduced in all subsequent trials. Initially, participants performed 3 min of baseline cycling at 40 W. Thereafter, the work rate increased at a rate of 20 W·min^−1^ until reaching the limit of tolerance. The test was completed when the pedal rate fell below 55 rpm despite verbal encouragement. The power output achieved at the point of exhaustion was recorded as the PPO. Blood glucose and lactate levels were measured at baseline and exhaustion respectively, and the HR was monitored throughout the test.

### 2.5. Interval cycling exercise protocol

The trial began 2.5 h after the consumption of beetroot juice to allow the timing to coincide with peak nitrate/nitrite bioavailability [54]. The participants warmed up for 5 min (cycling at 40 W at a self-selected pedal rate). Then, they performed the interval exercise (8 × 1-min bouts of cycling at 85% of PPO interspersed with 1-min active recovery at 20% of PPO), followed by 5-min cool-down cycling at 40 W at the same pedal rate. Given that this approach to interval training represents a midpoint with respect to intensity, recovery, and total volume, it is considered to be a “medium-volume” high-intensity interval exercise that is more appropriate for the general population [55]. All work intervals were preceded by a 3-s countdown with a “GO” command. Verbal encouragement and information pertaining to the interval number were provided during intermittent exercise.

### 2.6. Assessments of physiological responses

HR was monitored continuously through a heart rate sensor (Polar Team2 System, Polar Electro, Finland). Blood pressure was measured upon arrival (after a 15-min rest period in a seated position), pre-interval exercise, and post-interval exercise using an automatic sphygmomanometer (M7 Intelli IT, Omron, Japan). Blood glucose and lactate concentrations were measured using capillary blood samples from the fingertips with portable analyzers (Contour Plus Glucometer, Bayer Healthcare, Germany; Lactate Meter, Nova Biomedical Co., USA). Blood oxygen saturation (SpO_2_) was measured using a sensor placed on the fingertip (Yuwell YX306, Yuwell, China). Measurements of glucose, lactate, and SpO_2_ were taken four times, during the last 15 s of warm-up, within the first 15 s of recovery period 4, within the first 15 s of recovery period 8, and during the last 15 s of cool-down.

### 2.7. Assessments of psychological responses

Participants indicated their rating of perceived exertion (RPE) at the end of each stage using a 20-grade RPE scale (Borg scale) [56]. The validated scale was printed, and participants were able to visualize the scale when required.

Emotional arousal was measured before and after the exercise to describe the sentiments experienced during the trial using the 5-point Self-Assessment Manikin (SAM). This questionnaire used cartoons to reflect emotional experiences in terms of valence (positivity or negativity of emotion), arousal (intensity of emotion), and dominance (control over emotion) [57].

### 2.8. Sample size calculation

Sample size calculation was performed using G * POWER software (Heinrich-Heine-Universitӓt Düsseldorf, Germany) with an alpha of 0.05, a statistical power of 0.8 and a Cohen’s f value of 0.5 based on previous literature related to the effect of nitrate consumption on HR during interval exercise [39]. Accordingly, at least nine participants were required for the study. A plausible drop-out rate was set at 20%; therefore, 13 participants were recruited for this study.

### 2.9. Statistical analysis

All data were analyzed using SPSS software package (IBM SPSS version 26.0, Chicago, IL, USA). All continuous variables were presented as means ± SDs. The normality of the data was checked before further analysis. Data collected during the preliminary test and data for HR and RPE variables during the work intervals, recovery periods, and across the overall protocol in the main trials were analyzed using one-way analysis of variance (ANOVA). The Huynh – Feldt correction was applied when Mauchly’s sphericity test reached significance (*p* ≤ 0.05). The remaining data collected during the main trials were analyzed using a two-way (treatment × time) repeated-measures ANOVA to explore the effect of interventions (double-dose, single-dose, and placebo) over time on the magnitude of each dependent variable. A Bonferroni post hoc comparison was performed when ANOVA significance was reached. Partial eta squared ($\eta_{p}^{2}$) was used to indicate the magnitude of the difference among trials. Scores of 0.01, 0.06, and >0.14 were considered small, moderate, and large effect sizes, respectively [58]. Statistical significance was set at *p* ≤ 0.05.

## 3. Results

**Figure 2.**
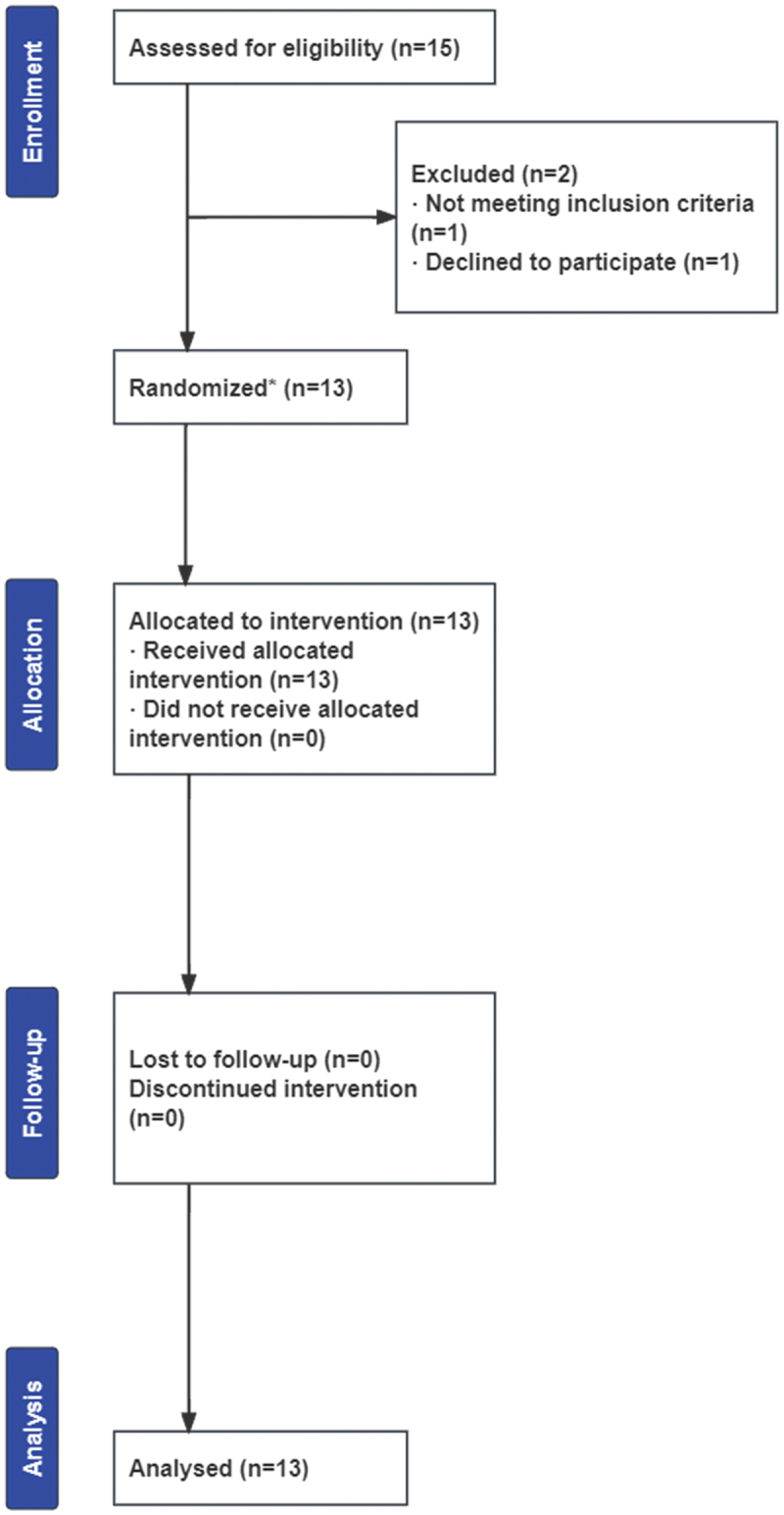
CONSORT flow diagram of study recruitment.

All participants completed three interval exercise trials and complied with the supplementation protocol. None of the participants withdrew from the study because of adverse responses to the nitrate supplementation or the exercise protocol. No illness was reported during the trial. A CONSORT flow diagram of study recruitment is shown in Figure 2.

*Randomization applies to the random allocation of beetroot juice containing 0 mmol (placebo), 6.45 mmol (single-dose), or 12.9 mmol (double-dose) ${NO}_{3}^{-}$.

### 3.1. Peak power output test

During the preliminary test, HR, RPE, blood glucose, and lactate levels at exhaustion were significantly different from those at baseline (Table 1). HR at exhaustion was 175 ± 10 beats/min, compared to 121 ± 18 beats/min at baseline (F[2.06, 24.68] = 177.99, *p* < .001, $\eta_{p}^{2}$ = .937). RPE at exhaustion was 18 ± 2, compared to 9 ± 2 at baseline (F [2,24] = 82.17, *p* < .001, $\eta_{p}^{2}$ = .873). Blood glucose at exhaustion was 5.0 ± 0.4 mmol/L, compared to 5.3 ± 0.6 mmol/L at baseline (F [1,12] = 4.94, *p* = .046, $\eta_{p}^{2}$ = .291). Blood lactate at exhaustion was 7.9 ± 2.4 mmol/L, compared to 2.4 ± 1.0 mmol/L at baseline (F [1,11] = 60.02, *p* < .001, $\eta_{p}^{2}$ = .845).

**Table 1.** HR, RPE, blood glucose, and lactate at baseline and exhaustion during the preliminary test.

	Baseline	Exhaustion
HR (beats/min)	121 ± 18	175 ± 10^†^
RPE	9.0 ± 1.9	17.9 ± 1.8^†^
Blood glucose (mmol/L)	5.3 ± 0.6	5.0 ± 0.4*
Blood lactate (mmol/L)	2.4 ± 1.0	7.9 ± 2.4^†^

HR = heart rate; RPE = rating of perceived exertion.

Data are presented as mean ± standard deviation; *Significantly different from baseline, *p* < .05; ^†^Significantly different from baseline, *p* < .001.

### 3.2. Effects of beetroot juice on interval cycling responses

#### 3.2.1. Assessments of physiological responses

The effects of different doses of nitrate supplementation on the HR are shown in Table 2. There was a main effect by time on HR during work intervals (F[2.13, 76.67] = 363.37, *p* < .001, $\eta_{p}^{2}$ = .910) but no interaction effect between treatment and time on HR during work intervals (F[4.26, 76.67] = 0.34, *p* = .862, $\eta_{p}^{2}$ = .018). The HR at the end of each work interval was significantly higher than that at the end of warm-up (all *p* < .001). The highest HR during work intervals (167 ± 11 beats/min) was observed in stage 8. Similarly, there was a main effect by time on HR during recovery periods (F[3.54, 127.52] = 65.74, *p* < .001, $\eta_{p}^{2}$ = .646) but no interaction effect between treatment and time on HR during recovery periods (F[7.08, 127.52] = 0.46, *p* = .865, $\eta_{p}^{2}$ = .025). The highest HR during the recovery period (148 ± 15 beats/min) was observed in Stage 7.

**Table 2.** Physiological and psychological responses to the interval exercise.

	Placebo	Single	Double	*p*-value
HR (beats/min)				
End warm-up	120 ± 20	116 ± 15	118 ± 18	.731
Work interval 1	150 ± 14	146 ± 12	147 ± 14	.948
Work interval 2	156 ± 13	152 ± 12	153 ± 14	.622
Work interval 3	161 ± 13	158 ± 10	156 ± 15	.164
Work interval 4	163 ± 12	160 ± 11	159 ± 12	.762
Work interval 5	164 ± 13	162 ± 10	162 ± 12	.667
Work interval 6	166 ± 13	164 ± 11	164 ± 12	.661
Work interval 7	167 ± 14	165 ± 11	166 ± 12	.681
Work interval 8	168 ± 12	166 ± 10	167 ± 12	.562
Recovery period 1	133 ± 18	129 ± 14	129 ± 17	.569
Recovery period 2	138 ± 17	134 ± 14	134 ± 18	.598
Recovery period 3	141 ± 17	138 ± 14	139 ± 17	.785
Recovery period 4	144 ± 16	139 ± 13	140 ± 14	.836
Recovery period 5	147 ± 16	143 ± 13	143 ± 15	.809
Recovery period 6	149 ± 16	144 ± 14	146 ± 16	.856
Recovery period 7	150 ± 17	147 ± 14	147 ± 14	.902
End cool-down	135 ± 15	134 ± 11	135 ± 14	.221
RPE				
End warm-up	9.0 ± 1.7	9.7 ± 1.8	9.4 ± 1.4	.785
Work interval 1	13.1 ± 1.9	13.1 ± 2.2	12.9 ± 2.2	.491
Work interval 2	14.0 ± 2.5	13.9 ± 2.0	14.1 ± 2.4	.622
Work interval 3	14.5 ± 2.1	14.5 ± 2.0	14.5 ± 2.5	.444
Work interval 4	15.4 ± 2.1	14.5 ± 2.1	14.9 ± 2.4	.682
Work interval 5	15.9 ± 2.3	15.4 ± 2.3	15.2 ± 2.3	.869
Work interval 6	16.0 ± 2.2	15.8 ± 1.9	15.7 ± 2.2	.711
Work interval 7	16.5 ± 2.2	16.1 ± 2.2	15.8 ± 2.0	.857
Work interval 8	16.3 ± 2.4	15.9 ± 2.4	16.2 ± 2.1	.759
Recovery period 1	10.0 ± 2.0	9.9 ± 1.9	9.5 ± 2.0	.905
Recovery period 2	10.2 ± 2.1	10.2 ± 1.8	9.9 ± 2.1	.482
Recovery period 3	10.9 ± 2.0	10.5 ± 1.9	10.8 ± 1.9	.852
Recovery period 4	11.3 ± 2.2	10.7 ± 1.8	10.6 ± 2.4	.622
Recovery period 5	11.8 ± 3.0	10.7 ± 2.0	10.5 ± 2.4	.418
Recovery period 6	11.9 ± 2.7	10.9 ± 2.0	10.9 ± 2.3	.464
Recovery period 7	12.3 ± 3.1	11.3 ± 2.2	11.1 ± 2.6	.546
End cool-down	10.5 ± 2.3	10.3 ± 2.2	10.3 ± 2.4	.886
Blood pressure (mmHg)				
Resting SBP	91 ± 7	89 ± 6	89 ± 7	.510
SBP pre-exercise	90 ± 8	88 ± 7	88 ± 6	.384
SBP post-exercise	96 ± 8	96 ± 8	99 ± 7	.919
Resting DBP	67 ± 5	61 ± 5	64 ± 4	.471
DBP pre-exercise	63 ± 5	61 ± 2	62 ± 4	.127
DBP post-exercise	68 ± 5	63 ± 5	66 ± 5	.978
Blood glucose (mmol/L)				
End warm-up	4.4 ± 0.7	4.5 ± 0.3	4.6 ± 0.7	.065
Recovery period 4	4.2 ± 0.6	4.3 ± 0.5	4.4 ± 0.4	.325
Recovery period 8	4.5 ± 0.6	4.2 ± 0.6	4.4 ± 0.3	.032
End cool-down	4.5 ± 0.5	4.4 ± 0.6	4.5 ± 0.3	.056
Blood lactate (mmol/L)				
End warm-up	2.5 ± 0.8	2.5 ± 0.7	2.8 ± 1.0	.458
Recovery period 4	5.7 ± 1.4	5.5 ± 1.0	5.3 ± 1.2	.326
Recovery period 8	7.3 ± 1.9	7.3 ± 1.5	7.1 ± 1.8	.847
End cool-down	6.8 ± 2.3	6.6 ± 1.6	6.7 ± 2.2	.281
SpO_2_ (%)				
End warm-up	98.2 ± 1.6	98.0 ± 1.1	99.3 ± 1.0	.022
Recovery period 4	98.0 ± 1.3	99.0 ± 1.1	98.4 ± 1.4	.551
Recovery period 8	97.8 ± 1.4	97.6 ± 1.2	97.9 ± 1.6	.875
End cool-down	97.7 ± 1.3	97.8 ± 1.3	97.9 ± 1.4	.951
Emotional arousal				
Pleasure pre-exercise	3 ± 1	3 ± 1	2 ± 1	.573
Pleasure post-exercise	2 ± 1	2 ± 1	2 ± 1	.262
Arousal pre-exercise	4 ± 1	4 ± 1	4 ± 1	.651
Arousal post-exercise	3 ± 1	3 ± 1	3 ± 1	.701
Dominance pre-exercise	3 ± 1	4 ± 1	4 ± 1	.909
Dominance post-exercise	4 ± 1	4 ± 1	3 ± 1	.054

HR = heart rate; RPE = rating of perceived exertion; SBP = systolic blood pressure; DBP = diastolic blood pressure; SpO_2_ = blood oxygen saturation; Placebo = placebo control group; Single = single-dose group; Double = double-dose group.

Data are presented as mean ± standard deviation; *p*-values of the Levene’s test for equality of variances are reported.

The mean HRs during work intervals, recovery periods, and across the overall protocol following nitrate supplementation are summarized in Table 3. Notably, mean HR during work intervals (F[1.90, 195.78] = 7.06, *p* = .001, $\eta_{p}^{2}$ = .064), recovery periods (F [2,180] = 9.89, *p* < .001, $\eta_{p}^{2}$ = .099), and across the overall protocol (F[1.93, 374.47] = 16.90, *p* < .001, $\eta_{p}^{2}$ = .080) was altered significantly by nitrate supplementation. Post hoc tests indicated that the HR was significantly lower in the single- and double-dose groups than in the control group during the work intervals, recovery periods, and across the overall protocol (Table 3). However, no significant differences were observed between the single- and double-dose groups at any time point (Table 3).

**Table 3.** HR and RPE variables during the work intervals, recovery periods, and across the overall protocol in the interval cycling exercise following nitrate supplementation.

	Placebo		Single		Double		Single vs Placebo		Double vs Placebo		Single vs Double	
	Mean	SD	Mean	SD	Mean	SD	MD [95% CI]	*p*	MD [95% CI]	*p*	MD [95% CI]	*p*
HR (beats/min)												
Work interval	162	14	159	12	159	14	3 [1−4]	.003	3 [0–5]	.013	0 [−2–2]	1.000
Recovery period	143	17	139	14	140	16	4 [2−6]	<.001	4 [1−6]	.001	0 [−2–1]	.639
Overall	153	18	150	17	150	18	3 [2−4]	<.001	3 [2−4]	<.001	0 [−1–1]	.772
RPE												
Work interval	15.2	2.4	14.9	2.3	14.9	2.4	0.3 [0.0–0.6]	.030	0.3 [0.0–0.6]	.051	0.0 [−0.3–0.3]	1.000
Recovery period	11.2	2.5	10.6	2.0	10.5	2.2	0.6 [0.3–1.0]	<.001	0.7 [0.4–1.1]	<.001	0.1 [−0.2–0.5]	.540
Overall	13.3	3.2	12.9	3.0	12.8	3.2	0.5 [0.2–0.7]	<.001	0.5 [0.3–0.7]	<.001	0.1 [−0.3–0.2]	.661

HR = heart rate; RPE = rating of perceived exertion; Placebo = placebo control group; Single = single-dose group; Double = double-dose group; SD = standard deviation; MD = mean difference; CI = confidence interval.

The effects of different nitrate supplementation doses on systolic (SBP) and diastolic (DBP) blood pressure are presented in Table 2. SBP and DBP at rest were not significantly different among the placebo control, single-, and double-dose groups. There was a main effect by time on both SBP (F [2,70] = 28.74, *p* < .001, $\eta_{p}^{2}$ = .451) and DBP (F [2,70] = 9.34, *p* < .001, $\eta_{p}^{2}$ = .211). Post hoc tests indicated that SBP at rest (90 ± 7 mm Hg) and pre-exercise (89 ± 7 mm Hg) were significantly lower than SBP post-exercise (97 ± 8 mm Hg; both *p* < .001). DBP pre-exercise (62 ± 4 mm Hg) was significantly lower than DBP post-exercise (66 ± 5 mm Hg; *p* < .001). However, there was no interaction effect between treatment and time on either SBP (F [4,70] = 0.88, *p* = .480, $\eta_{p}^{2}$ = .048) or DBP (F [4,70] = 0.84, *p* = .503, $\eta_{p}^{2}$ = .046).

The effects of different doses of nitrate supplementation on blood glucose and lactate concentrations are shown in Table 2. ANOVA analyses revealed that there was neither a main effect by time (F[1.77, 63.76] = 2.42, *p* = .104, $\eta_{p}^{2}$ = .063) nor an interaction effect between treatment and time on blood glucose (F[3.54, 63.76] = 1.08, *p* = .370, $\eta_{p}^{2}$ = .057). In contrast, there was a main effect by time on blood lactate concentration (F[2.43, 87.54] = 135.14, *p* < .001, $\eta_{p}^{2}$ = .790). Post hoc tests revealed that blood lactate levels were significantly higher during recovery periods 4 (*p* < .001) and 8 (*p* < .001) and the end of cool-down (*p* < .001) relative to the end of warm-up. Specifically, blood lactate increased by ~ 1.8-fold during recovery period 8 compared to that at the warm-up stage. There was, however, no interaction effect between treatment and time on blood lactate concentration (F([4.86, 87.54] = 0.29, *p* = .913, $\eta_{p}^{2}$ = .016). In terms of SpO_2_, there was a main effect by time (F [3,96] = 3.57, *p* = .017, $\eta_{p}^{2}$ = .100) but no interaction effect between treatment and time (F [6,96] = 1.39, *p* = .228, $\eta_{p}^{2}$ = .080). Post hoc tests revealed that SpO_2_ was significantly lower during recovery period 8 relative to the end of warm-up and recovery period 4 (*p* < .05) and that SpO_2_ was significantly lower at the end of cool-down relative to recovery period 4 (*p* < .05).

#### 3.2.2. Assessments of psychological responses

The effects of different nitrate supplementation doses on RPE are shown in Table 2. There was a main effect by time on RPE at work intervals (F[3.41, 122.67] = 155.53, *p* < .001, $\eta_{p}^{2}$ = .812), indicating that RPE increased significantly across the exercise session in all trials. However, there was no interaction effect between treatment and time on RPE during work intervals (F[6.82, 122.67] = 0.70, *p* = .666, $\eta_{p}^{2}$ = .038). The highest RPE at work interval (16 ± 2) was reported in stage 8. Similarly, there was a main effect by time on RPE during recovery periods (F[3.91, 140.74] = 14.73, *p* < .001, $\eta_{p}^{2}$ = .290) but no interaction effect between treatment and time on RPE during recovery periods (F[7.82, 140.74] = 1.00, *p* = .436, $\eta_{p}^{2}$ = .053).

The mean RPE values during work intervals, recovery periods, and across the overall interval exercise protocol following nitrate supplementation are summarized in Table 3. Specifically, RPE during recovery periods (F [2,180] = 10.39, *p* < .001, $\eta_{p}^{2}$ = .104), and across the overall protocol (F [2,388] = 11.96, *p* < .001, $\eta_{p}^{2}$ = .058) was altered significantly by nitrate supplementation. Post hoc tests indicated that RPE was significantly lower in the single- and double-dose groups than in the control group during recovery periods, and across the overall protocol (Table 3). However, there was no significant difference between the single and double doses at any time point (Table 3).

There was a main effect by time on rating of pleasure (F [1,36] = 9.56, *p* = .004, $\eta_{p}^{2}$ = .210) and arousal (F [1,36] = 18.24, *p* < .001, $\eta_{p}^{2}$ = .336), but no interaction effect between treatment and time on rating of pleasure (F [2,36] = 0.05, *p* = .952, $\eta_{p}^{2}$ = .003) or arousal (F [2,36] = 0.55, *p* = .583, $\eta_{p}^{2}$ = .030). Generally, participants reported higher pleasure and arousal ratings following the interval exercise. There was neither a main effect by time on rating of dominance (F [1,36] = 0.00, *p* = 1.000, $\eta_{p}^{2}$ = .000) nor an interaction effect between treatment and time on rating of dominance (F [2,36] = 3.17, *p* = .054, $\eta_{p}^{2}$ = .150).

## 4. Discussion

The purpose of the present study was to investigate the acute dose–response effects of nitrate supplementation on physiological and psychological responses to high-intensity interval exercise in women. Specifically, we studied how acute ingestion of beetroot juice with three different nitrate contents (i.e. 0 mmol ${NO}_{3}^{-}$/140 mL, 6.45 mmol ${NO}_{3}^{-}$/140 mL, and 12.9 mmol ${NO}_{3}^{-}$/140 mL) impacted HR, BP, blood glucose, and lactate levels, SpO_2_, RPE, and emotional arousal during high-intensity interval cycling. Our principal findings were that significant decreases in the mean HR and RPE during work intervals, recovery periods, and across the overall protocol were evident after the acute consumption of beetroot juice containing 6.45 mmol and 12.9 mmol ${NO}_{3}^{-}$, with no further benefits observed with the beetroot juice containing 12.9 mmol ${NO}_{3}^{-}$.

Consistent with a previous study [39], the mean HR was lower during intermittent exercise following nitrate supplementation than in the control. However, in contrast to our experimental hypothesis, no further effect on HR was evident following a higher dose of nitrate supplementation, suggesting a “saturation” effect. Nyakayiru et al. [39] reported a significant decrease in the mean HR for a high-intensity intermittent running test following a 6-day supplementation period. The nitrate dose ingested by the participants in the experimental group per day (~12.9 mmol ${NO}_{3}^{-}$) was equal to the double-dose group in the present study. In addition, the magnitude of benefits in lowering the mean HR during the high-intensity intermittent-type exercise reported by Nyakayiru et al. with chronic supplementation (mean difference = 3 beats/min, *p* = .014) was comparable to that of observed in the present study with acute supplementation. It has been suggested that the reduction in HR following nitrate supplementation may be a function of improved contractility of the left ventricle, which consequently allows for an increase in stroke volume [59,60]. As a result, HR would be lower while the cardiac output is still maintained at the same level. While a reduction of 3 bpm in HR during exercise may considered modest for the general population, it could still hold significant relevance for more well-trained individuals and elite athletes, who have limited margins for gaining a performance advantage. From a practical perspective, even this slight reduction in HR may be associated with improved exercise tolerance, as indicated by lower RPE during work intervals, recovery periods, and across the overall protocol with acute nitrate supplementation compared to the control condition in the present study. Although RPE is a measure based on subjective feelings, it can imply that the participants felt more comfortable when performing the exercise at the same intensity.

Our results revealed a main effect by time on both SBP and DBP, but there was no interaction effect between treatment and time. When comparing our findings to those in the existing literature, Thompson et al. [40,61] reported a reduction in SBP with a chronic supplementation, while others reported no significant difference in blood pressure after chronic nitrate supplementation [32,35]. Wylie et al. [50] reported significant interaction effects on SBP following acute consumption of 4.2, 8.4, and 16.8 mmol ${NO}_{3}^{-}$. The inconsistency in the efficacy of nitrate supplementation in lowering the blood pressure could be partly attributed to the timing of blood pressure measurement and sex differences in baseline BP. According to Wylie et al. [50], a peak reduction in SBP was observed at 4 h after consumption of 4.2 (~5 mm Hg), 8.4 (~9 mm Hg), and 16.8 (~10 mm Hg) mmol ${NO}_{3}^{-}$, whereas we measured BP only at 2.5 h and 3 h after consumption. Meanwhile, compared to Wylie et al. [50], who recruited only men, all participants recruited in our study were women. Moreover, the baseline BP measured in our study (approximately 90/64 mm Hg) was substantially lower than the baseline BP reported by Wylie et al. (approximately 120/68 mm Hg), suggesting less room for noticeable changes. Therefore, despite the beneficial effects of nitrate-rich beetroot juice observed in prior studies [32,35,40,50,61] on blood pressure regulation and cardiovascular health, the result of the present study only indicated a cardiovascular response to the cycling exercise over time with no significant differences among treatment groups.

In accordance with a previous study [30], our results demonstrated that nitrate supplementation did not affect the increase in blood lactate concentration from baseline to the end of interval exercise under the 1-min work-interval protocol. Interestingly, Wylie et al. [30] reported in the same study that the increase in blood lactate was greater under the 6-s and 30-s work-interval protocols at supramaximal intensity in the group with nitrate supplementation than in the placebo control group. A possible explanation for this discrepancy might be the difference in exercise intensity (i.e. supramaximal versus near-maximal), with supramaximal exercise recruiting more anaerobic type II muscle fibers. Consequently, an increase in muscle hypoxia and acidity facilitates NO biosynthesis [62,63], eventually leading to an increase in physiological responses (e.g. an increase in blood lactate) [16,23,64]. Conversely, the effect of nitrate supplementation on lactate responses to the near-maximal exercise (e.g. protocol used in our study) may have been less pronounced than that to the supramaximal exercise.

It is worth noting that the present study did not assess any performance outcome, as previous studies have already extensively focused on performance-based measure by including peak power output, mean power output, time-trial performance, and time-to-exhaustion as their primary outcomes. One recent systematic review with meta-analysis [41] has concluded that nitrate supplementation could improve exercise performance, in particular, in sessions lasting between 2 and 10 mins and that ingestion of 5–14.9 mmol ${NO}_{3}^{-}$ taken ≥150 min prior to exercise appears optimal for performance gains. Therefore, our research interest was more in the physiological and psychological responses to interval exercise after nitrate supplementation rather than the athletic performance. In addition, since the participants in our study were recreationally active young women, who do not look for an improvement in performance during competition, hence, we measured outcomes that were closer to real life setting (e.g. perceived exertion, emotional arousal). The underlying motivation was to figure out whether nitrate supplementation would lower people’s perceived discomfort and become more engaged/aroused during exercise, as traditional high-intensity exercise is hypothesized to have a lower exercise adherence due to the greater displeasure caused by higher intensity exercise above the ventilatory threshold [65].

Our study contributes to the limited body of research investigating the effects of nitrate supplementation in women. Furthermore, the present study provides new insights into the efficacy of nitrate supplementation in alleviating perceived effort during interval exercise, highlighting the practical benefits of this supplement. However, our study has a few limitations. Our sample size calculation was based on the primary outcome of the HR effect, which may have resulted in insufficient statistical power to detect the dose–response relationship on some secondary outcomes due to the relatively small sample size. Another limitation is the lack of measurement of plasma nitrate and nitrite concentrations following acute consumption of beetroot juice. Future studies should consider larger sample sizes to detect potential differences between groups and examine how fluctuations in plasma or muscle nitrate and nitrite concentrations would affect the physiological responses to interval exercise. Furthermore, only oral contraceptives intake was included as the exclusion criteria in the present study. Although none of the participants in our study had used any form of contraceptives, it is encouraged that future studies should consider excluding women applying other forms of contraceptives, for example, contraceptive patches, that control endogenous sex hormones concentrations as oral contraceptives. Finally, during postprandial resting period, although participants were only allowed to consume plain water, we did not control for the amount of water intake. Future study should consider recording and monitoring the water intake to ensure the consistency of water consumption among participants.

## 5. Conclusions

In conclusion, our study demonstrated that acute nitrate ingestion in the form of beetroot juice significantly decreased the mean HR and RPE during work intervals, recovery periods, and across the overall protocol in women. However, additional benefits for these parameters were not evident after a higher dose of acute beetroot juice supplementation.

## Data Availability

Datasets used in this study are available from the corresponding author under reasonable request.

## Publication statement

This manuscript has not been published elsewhere.
